# Supplementary material for: A prediction model of PTSD in the Israeli population in the aftermath of october 7th, 2023, terrorist attack and the Israel–Hamas war
Source: Isr J Health Policy Res. 2024 Oct 29;13:63. doi: 10.1186/s13584-024-00644-6 (PMC11520871; doi:10.1186/s13584-024-00644-6)
Supplement: Supplementary file 1 — Supplementary Material 1. [file 13584_2024_644_MOESM1_ESM.docx]

**Appendix 1-** Numerical estimations for exposure groups:

| Group | Total | Group population estimation |
| --- | --- | --- |
| **1. Direct exposure to the October 7th terror attacks** | 39,664 | Participants of music festivals and citizens of communities who were subject to the terror attack of Hamas perpetrators.  Communities included: ^[[1]](#footnote-1)^ ^[[2]](#footnote-2)^  Participants of music festivals – 3600^[[3]](#footnote-3)^ ^[[4]](#footnote-4)^  Communities and cities in the Gaza envelope area which were invaded:   1. Mishor Hagefen neighborhood, Ofaqim – 3509 (1100 households^[[5]](#footnote-5)^ * 3.19 people per household in Israel^[[6]](#footnote-6)^) 2. Sderot – 17647 (Sderot city was attacked in several central places^[[7]](#footnote-7)^ ^[[8]](#footnote-8)^ ^[[9]](#footnote-9)^. In the absence of accurate information about the number of neighborhoods within the city that were attacked, half of the city was included). 3. Be’eri - 1071 4. Ein Habsor - 1109 5. Nirim - 416 6. Mivtahim – 455 7. Mefalsim - 1,057 8. Pri Gan -249 9. Magen – 540 10. Yad Mordechai -830 11. Nir Yitzhak - 633 12. Erez - 616 13. Re’im – 422 14. Kissufim - 294 15. Sa’ad – 838 16. Alumim -531 17. Nir Am – 726 18. Kerem Shalom - 220 19. Zikim -918 20. Yakhini -730 21. Ein HaShlosha – 353 22. Sufa - 233 23. Nir Oz – 380 24. Holit -210 25. Netiv HaAsara - 948 26. Kfar Aza -787 27. Nahal Oz – 479 28. Gevim - 619   The music festivals' participants (364)^[[10]](#footnote-10)^ and 392 civilians^[[11]](#footnote-11)^ (including civilian emergency squad) who were killed, were subtracted. |
| **2. Extreme proximity to the October 7th terror attacks** | 121,061  After subtraction | Communities and neighborhoods in this group were not infiltrated by Hamas perpetrators, but their residents were under severe perceived life threat due to the notion of the invasion to nearby areas, and the high probability of the perpetrators invading their homes. These communities include Gaza envelope settlements^[[12]](#footnote-12)^, The city of Netivot (50,766) Ofaqim city (36,723)^[[13]](#footnote-13)^ (sum of 157,489). Civilians from 1 group were subtracted. |
| **3. Soldiers in combat and support units involved in the war** | 144,227 after subtraction | Soldiers of 10 divisions in the escalation areas - Gaza Strip, the Israel-Lebanon border, and the West Bank (sum of 165,000). Divisions numbers: 36^[[14]](#footnote-14)^, 252^[[15]](#footnote-15)^, 143(Gaza division)^[[16]](#footnote-16)^ ^[[17]](#footnote-17)^, 162^[[18]](#footnote-18)^, 99^[[19]](#footnote-19)^, 98^[[20]](#footnote-20)^, 210^[[21]](#footnote-21)^, 91^[[22]](#footnote-22)^, 877(Judea and Samaria division)^[[23]](#footnote-23)^, 146^[[24]](#footnote-24)^.  The size of a division ranges from 13,000 to 20,000 soldiers, calculated with an average of 16,500^[[25]](#footnote-25)^.  We assumed that the geographical dispersion of soldiers is similar to the general population. As mentioned, in case of multifaceted exposure, we chose the higher prevalence rate. As some soldiers reside in regions exhibiting elevated PTSD risk compared to their military service risk, (i.e., groups 1, 2,4), their relative parts of the Israeli population (group 1 – 0.41%, 2 – 1.24%, 4 – 10.94%) have been subtracted from the soldiers' group and classified in the citizens' groups (670, 2,045 and 18,058 soldiers respectively, sum of 20,773 soldiers). |
| **4. Civilians under intense exposure to rocket attacks (living up to 40 km from the Gaza Strip)** | 1,069,011 after subtraction | Civilians who live in the cities and communities located up to 40 km from the Gaza-Israel border3 ^[[26]](#footnote-26)^.  All of the relevant communities and cities are listed, but those included in categories 1 and 2 were subtracted.    Citizens (sum of 1229736, regional councils are written in uppercase)-   1. Sederot - 35295 2. SHA'AR HANEGEV – 9900 3. Netivot – 50765 4. Ashqelon – 159836 5. SEDOT NEGEV – 11474 6. RAMAT NEGEV – 8679 7. HOF ASHQELON – 19828 8. Qiryat Gat – 66853 9. ESHKOL – 15679 10. Ofaqim – 36723 11. SHAFIR – 13875 12. MERHAVIM – 15018 13. Rahat – 81836 14. YO'AV – 9290 15. BE'ER TOVIYYA – 23603 16. Ashdod – 227392 17. LAKHISH – 14237 18. Qiryat Mal'akhi – 26197 19. Lehavim – 7376 20. Bene Ayish – 6771 21. GEDEROT – 5193 22. Gedera – 32189 23. Be'er Sheva – 215703 24. Laqye – 16868 25. NAHAL SOREQ – 10286 26. BENE SHIM'ON – 11710 27. HEVEL YAVNE – 7055 28. Gan Yavne – 25147 29. Yavne – 57261 30. Omer – 7682 |
| 5. **Civilians under moderate exposure to rocket attacks (living 40-80 km from the Gaza Strip** | 4,960,469 after exclusion | Civilians who live between 40-80 kilometers from the Israel-Gaza border 3 26.  Given the geographical dispersion of soldiers residential areas throughout Israel, some of them live 40 to 80 km from the Gaza Strip, an area with lower PTSD risk compared to their military service risk. Therefore, their relative part of the Israeli population (51.66%) was assumed to be their relative part of the soldiers' group and has been subtracted from group 5 and classified in the soldiers' group (sum of 85,234 soldiers).  Civilians (sum of 5,045,703, regional councils are written in uppercase)-  1. Tel Sheva - 23,486  2. Metar - 11,065  3. Qiryat Eqron - 10,975  4. Al-Kasum - 18,995  5. Mazkeret Batya - 15,969  6. Hura - 25,676  7. Rehovot - 152,576  8. Segev-Shalom - 13,010  9. MATTE YEHUDA - 51,131  10. Bet Shemesh - 165,582  11. Nes Ziyyona - 50,150  12. GEZER - 27,833  13. Be'er Ya'aqov - 33,324  14. Rishon LeZiyyon - 264,001  15. Ramla - 81,498  16. Qiryat Arba - 7,563  17. Bat Yam - 129,442  18. GAN RAWE - 6,659  19. Holon - 197,672  20. Zur Hadassa - 12,091  21. Lod - 86,968  22. GUSH EZYON - 27,355  23. Betar Illit - 67,141  24. Bet Dagan - 7,859  25. Azor - 13,386  26. Modi'in-Makkabbim-Re'ut - 102,282  27. Efrat - 11,942  28. HAR HEVRON - 11,162  29. Qiryat Ye'arim - 6,716  30. Kuseife - 25,112  31. Or Yehuda - 40,038  32. Abu Ghosh - 8,029  33. Yehud-Monoson - 31,672  34. Giv'atayim - 62,226  35. MATTE BINYAMIN - 77,098  36. Tel Aviv - Yafo - 479,152  37. Ramat Gan - 175,612  38. Shoham - 23,729  39. Qiryat Ono - 44,242  40. Har Adar - 4,145  41. Savyon - 3,960  42. Modi'in Illit - 85,643  43. Mevasseret Ziyyon - 25,819  44. Ganne Tiqwa - 24,515  45. Yeroham - 11,394  46. Giv'at Shemu'el - 29,322  47. HEVEL MODI'IN - 23,923  48. Bene Beraq - 221,768  49. SEDOT DAN - 15,999  50. Petah Tiqwa - 257,388  51. El'ad - 50,191  52. Jerusalem - 995,073  53. Giv'at Ze'ev - 22,281  54. Dimona - 37,513  55. Arad - 29,319  56. Ramat HaSharon - 48,091  57. Rosh HaAyin - 75,187  58. Herzliyya - 108,955  59. Hod HaSharon - 66,007  60. Kefar Shemaryahu - 1,954  61. Kafar Qasem - 25,775  62. Ma'ale Adummim - 38,073  63. Ra'annana - 81,434  64. Kafar Bara - 4,048  65. HOF HASHARON - 15,394  66. Jaljulye - 10,824  67. Oranit - 9,385  68. Kefar Sava - 101,440  69. Bet El - 6,315  70. Elqana - 4,421  71. BRENNER - 8,065  72. TAMAR - 1,625 |
| 6. **Indirectly affected communities (living more than 80 km from the Gaza Strip)** | 3,433,286 after exclusions | Civilians who live in communities and cities located more than 80 km from the Gaza-Israel border  (sum of 3,492,279)3 26.  Given the geographical dispersion of soldiers' residential areas throughout Israel, some of them more than 80 km from the Gaza Strip, an area with lower PTSD risk compared to their military service risk. Therefore, their relative part of the Israeli population (35.75%) was assumed to be their relative part of the soldiers' group, and has been subtracted from group 6 and classified in the soldiers’ group (sum of 58,993 soldiers).  This group includes the rest of the Israeli population (sum of 9,767,718 residents3), which is not included in the other groups. |

1. Data N12 (2023). All the settlements, all the stories: this is what the days of fighting in the Gaza Envelope looked like. *Mako*. https://www.mako.co.il/news-specials/data_n12/Article-3458498d1cd3b81027.htm [↑](#footnote-ref-1)
2. Israel Central Bureau of Statistics (2022). *Regional statistics.* <https://www.cbs.gov.il/he/publications/doclib/2019/ishuvim/bycode2022.xlsx> [↑](#footnote-ref-2)
3. Almog, T (2024, January 1^st^). Victims of the Nova party demand hundreds of millions from the state. *Kan*. https://www.kan.org.il/content/kan-news/local/667175/ [↑](#footnote-ref-3)
4. Tzalah, Z. (2023, October 10^th^). 100 People participated in 'Psyduck', a small party between Nirim and Nir Oz. 10 of them are missing. *Mako.* https://www.mako.co.il/music-news/Article-551663c5e1a1b81026.htm [↑](#footnote-ref-4)
5. The Day After Coalition (2022, May). *Mapping the challenge – Ofaqim - Kibbutz Galuyot and Mishor HaGefen neighborhoods.* https://negevgalil.org.il/wp-content/uploads/2022/05/2-%D7%9E%D7%99%D7%A4%D7%95%D7%99-%D7%94%D7%90%D7%AA%D7%92%D7%A8-%D7%A9%D7%9B%D7%95%D7%A0%D7%AA-%D7%A7%D7%99%D7%91%D7%95%D7%A5-%D7%92%D7%9C%D7%95%D7%99%D7%95%D7%AA-%D7%90%D7%95%D7%A4%D7%A7%D7%99%D7%9D.pdf [↑](#footnote-ref-5)
6. *Households* (n.d.). Israel Central Bureau of Statistics. Retrieved February 8^th^, 2024, from https://www.cbs.gov.il/en/subjects/Pages/Households.aspx [↑](#footnote-ref-6)
7. Heller, S. (2023, October 18^th^). “The helplessness cannot be forgotten": The moments of fear from the roof in Sderot. *Walla.* https://news.walla.co.il/item/3617078 [↑](#footnote-ref-7)
8. Green-Shaulov, R. (2023, November 9^th^). The last photo of holocaust survivors - Moments before the massacre in Sderot: 'They killed them all'. *Ynet.* https://www.ynet.co.il/news/article/ryi5jt9mp [↑](#footnote-ref-8)
9. Glikman, E. (2023, October 8^th^). The police station was destroyed, concerns about terrorists still remain: Sderot turns into a battlefield. *Ynet.* https://www.ynet.co.il/news/article/s1clfylb6 [↑](#footnote-ref-9)
10. Tegania, B. (2023, November 17^th^). The police investigation reveals: 364 were slaughtered at the Re’im Party. *Mako.* [https://www.mako.co.il/news-military/6361323ddea5a810/Article-ec3f8e05fcedb81027.htm](https://www.mako.co.il/news-military/63613א23ddea5a810/Article-ec3f8e05fcedb81027.htm) [↑](#footnote-ref-10)
11. Hovel, R )2024, January 18^th^). " There are two abductions you haven't heard about": Interview with the one who follows them. *Shakoof.* https://shakuf.co.il/46990 [↑](#footnote-ref-11)
12. The iCenter for Israel Education (2023, October). *FAQ: Gaza and Israel*. https://theicenter.org/wp-content/uploads/2023/10/FAQ-Gaza-and-Israel.pdf [↑](#footnote-ref-12)
13. Israel Central Bureau of Statistics (2024, January 31th). *Population.* https://www.cbs.gov.il/he/publications/LochutTlushim/2020/%D7%90%D7%95%D7%9B%D7%9C%D7%95%D7%A1%D7%99%D7%99%D7%942020.xlsx [↑](#footnote-ref-13)
14. Israel Defense Forces. (2023, November 5^th^). *36th Division operates in Northern Gaza, reaches position along Gaza's Coast.* https://www.idf.il/en/mini-sites/idf-press-releases-regarding-the-hamas-israel-war/november-23-pr/36th-division-operates-in-northern-gaza-reaches-position-along-gaza-s-coast/ [↑](#footnote-ref-14)
15. Israel Defense Forces. (2023, November 18^th^). *An entire reserve division is maneuvering in the Gaza Strip.* https://www.idf.il/148209 [↑](#footnote-ref-15)
16. Zeitoon, Y. (2023, October 12^th^). Gaza Brigade Commander in first statement: 'We stopped the attack. We did everything to fight'*.* *Ynet*. https://www.ynet.co.il/news/article/bjjhjssza [↑](#footnote-ref-16)
17. Israel Defense Forces. (2024, January 30^th^). *Chief of Staff to reservists in Gaza Brigade: 'There will be no refuge for terror in Gaza, in the West Bank, in Lebanon, neither above ground nor in tunnel exits'".* https://www.idf.il/177294 [↑](#footnote-ref-17)
18. Israel Defense Forces. (2023, December 19^th^). *Explosives, missiles, and rockets; Troops identified weapons production sites and a large stockpile of weapons.* https://www.idf.il/165238 [↑](#footnote-ref-18)
19. Israel Defense Forces. (2023, December 21^th^). *Special operation: 99th Division fighters expand IDF activity in the Central Gaza Strip.* https://www.idf.il/164715 [↑](#footnote-ref-19)
20. Israel Defense Forces. (2024, January 29^th^). *Combat in Khan Yunis: 98th Brigade operations above and below ground.* https://www.idf.il/178119 [↑](#footnote-ref-20)
21. Israel Defense Forces. (2024, January 3^rd^). *"We are very strongly prepared in the North, focusing on combatting Hamas".* https://www.idf.il/169079 [↑](#footnote-ref-21)
22. Israel Defense Forces. (2023, December 10th). *The Chief of the General Staff conducts situational assessment in the 91st Division.* https://www.idf.il/161185 [↑](#footnote-ref-22)
23. Ben Gigi, N (2024, January 10^th^). *100 days of war on terror - The data behind the activity in Judea and Samaria revealed.* Israel Defense Forces. https://www.idf.il/171211 [↑](#footnote-ref-23)
24. Israel Defense Forces. (2023, December 20^th^). *"We're not going back to what was before".* https://www.idf.il/164429 [↑](#footnote-ref-24)
25. Division. (2024, January 2). In *HaMichlol*.https://www.hamichlol.org.il/%D7%90%D7%95%D7%92%D7%93%D7%94 [↑](#footnote-ref-25)
26. The Ministry of Welfare and Social Affairs (n.d.). *List of settlements and their distance from the border.* https://www.molsa.gov.il/Subsidizing/Documents/%D7%9E%D7%95%D7%A1%D7%93%D7%95%D7%AA%20%D7%A6%D7%99%D7%91%D7%95%D7%A8/2015/%D7%99%D7%A9%D7%95%D7%91%D7%99%D7%9D%20%D7%95%D7%9E%D7%A8%D7%97%D7%A7%D7%9D%20%D7%9E%D7%94%D7%92%D7%91%D7%95%D7%9C.xls [↑](#footnote-ref-26)
