# Supplementary material for: A prediction model of PTSD in the Israeli population in the aftermath of october 7th, 2023, terrorist attack and the Israel–Hamas war
Source: Isr J Health Policy Res. 2024 Oct 29;13:63. doi: 10.1186/s13584-024-00644-6 (PMC11520871; doi:10.1186/s13584-024-00644-6)
Supplement: Supplementary file 2 — Supplementary Material 2. [file 13584_2024_644_MOESM2_ESM.docx]

**SUPPLEMENTARY MATERIAL**

**Omission of studies in meta-analyses**

**Group 1: Direct exposure to the October 7th terror attacks**.

In the systematic review by Paz García-Vera, et al. (2016), one study (Ankri, et al., 2010) was omitted as it included help-seeking population.

**Group 3: Soldiers in combat and support units involved in the war.**

In the review by Richardson, et al. (2010) studies were omitted for the following reasons: 18 studies included questionnaire assessments or non-clinical assessments (Barrett et al., 2002; Browne et al., 2007; Dohrenwend et al., 2006; Eisen et al., 2004; Goldberg et al., 1990; Goss-Gilroy 1998; Gray et al., 2002; Hoge et al., 2004; Hoge et al., 2006; Hotopf et al., 2006; Iowa Persian Gulf Study Group, 1997; Iversen et al., 2008; Jones et al., 2006; Kang et al., 2003; Smith et al., 2008; Stretch et al., 1993; Thompson et al., 2006; Unwin et al., 1999; Wolfe et al., 1999); two studies re-analyzed existing data from another sample (Dohrenwend et al., 2006; Thompson et al., 2006); one study included help-seeking population (Lee et al., 2002); and one study that did not examine current PTSD prevalence (Ikin, et al., 2004).

In the review by Xue, et al., (2015), studies were omitted for the following reasons: 23 studies included questionnaire assessments or non-clinical assessments (Barrett et al., 2002; Booth-Kewleyet al., 2010; Du Preez et al., 2011; Goldmann et al., 2012; Harbertson et al., 2013; Iversen et al., 2008; Iversen et al., 2009; Jones et al., 2006; Jones et al., 2012; Kline et al., 2013; Koenen et al., 2003; LeardMann et al., 2009; LeardMann et al., 2010; Macera et al., 2014; MacGregor et al., 2013; McCarren et al., 1995; Phillips et al., 2010; Riviere et al., 2011; Rona et al., 2007; Rona et al., 2012; Sandweiss et al., 2011; Van Liempt et al., 2013; Wells et al., 2012); three studies examined PTSD diagnosis in medical files without specific reference to the war exposure (Mayo, et al., 2013; Maguen, et al., 2012; MacGregor, et al., 2012); and three studies did not examine current prevalence (Tracie, et al., 2013; Dohrenwend, et al., 2008; Koenen, et al., 2002).

**Group 4: Civilians under intense exposure to rocket attacks (living up to 40 km from the Gaza Strip)**.

In the review by Greene, et al., 2018, out of the twelve studies that presented included PTSD prevalence estimates, studies were omitted for the following reasons: two studies examined PTSD within the timeframe of war exposure (Besser and Neria, 2010; Nuttman-Shwartz, et al., 2015); one study did not specify whether measurement was conducted during or after war exposure (Nuttman-Shwart, et al., 2014); and two studies did not clarify whether they assessed PTSD symptoms that related specifically to rocket attacks (Ben-Ezra et al., 2015; Stein, et al., 2013).

**Group 6: Indirectly affected communities** (**living more than 80 km from the Gaza strip)**.

In the review by Paz García-Vera (2016), studies were omitted for the following reasons: six studies did not distinguish between people who were directly affected by the terror attack and those who were not (Miguel-Tobal, et al., 2005; Nandi, et al., 2005; Lawyer, et al., 2006; Galea, et al., 2004; Boscarino, et al., 2004; Stuber, et al., 2006); one study included help-seeking population (Shear, et al., 2006); one study included a community not affected by the terror attack (Henrikson, et al., 2010); and one study was conducted within a period of marked threat of terrorism (Hobfall, et al., 2011).

**Forest plots of meta-analyses**

Direct exposure to terror attacks meta-analysis


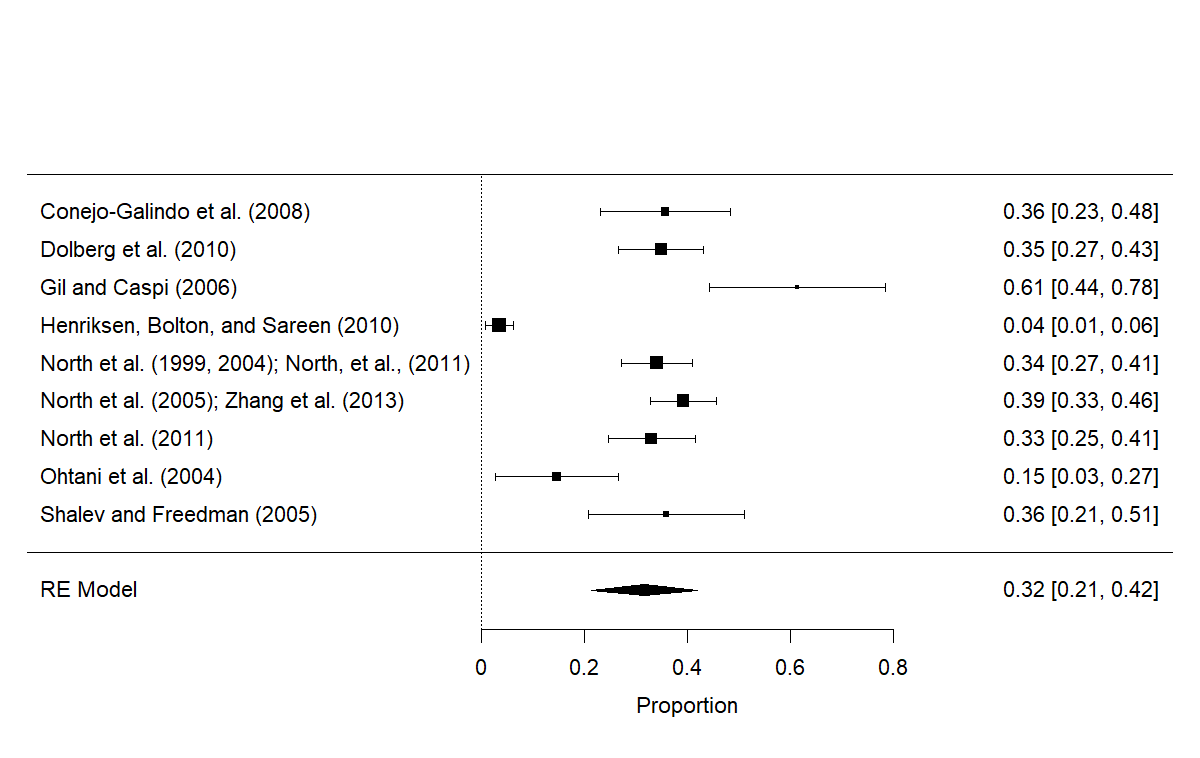


Soldiers meta-analysis


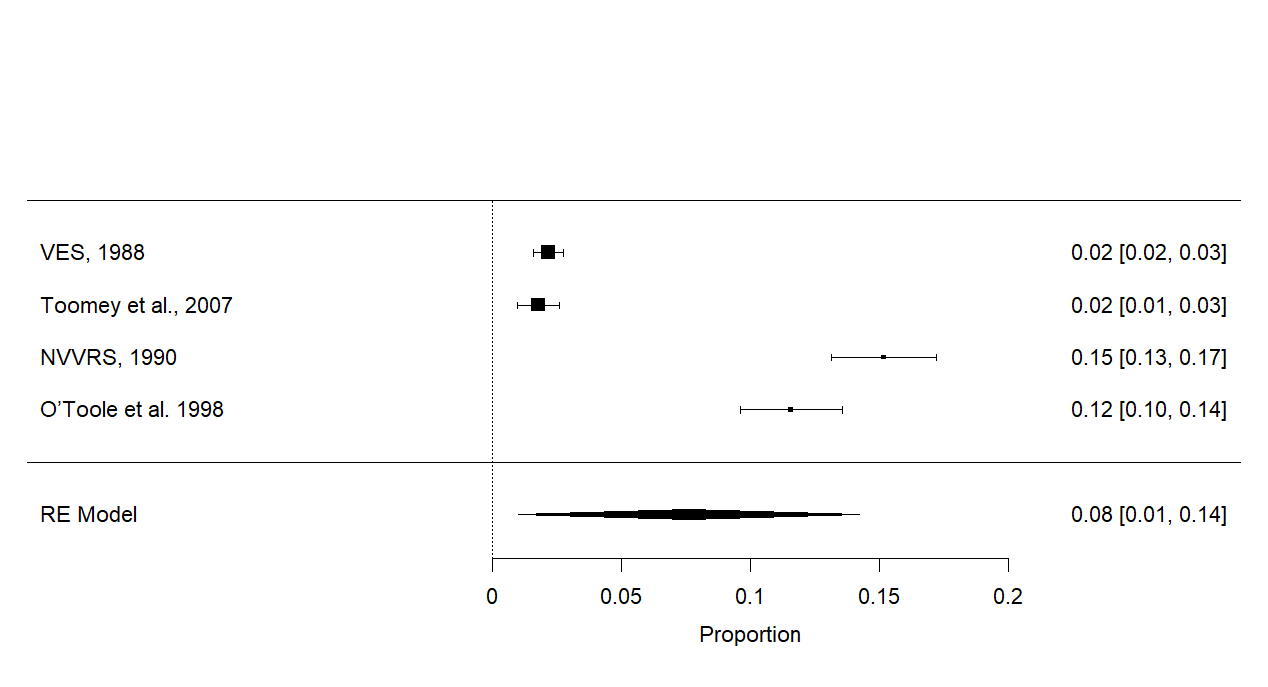


Intense rocket exposure meta-analysis


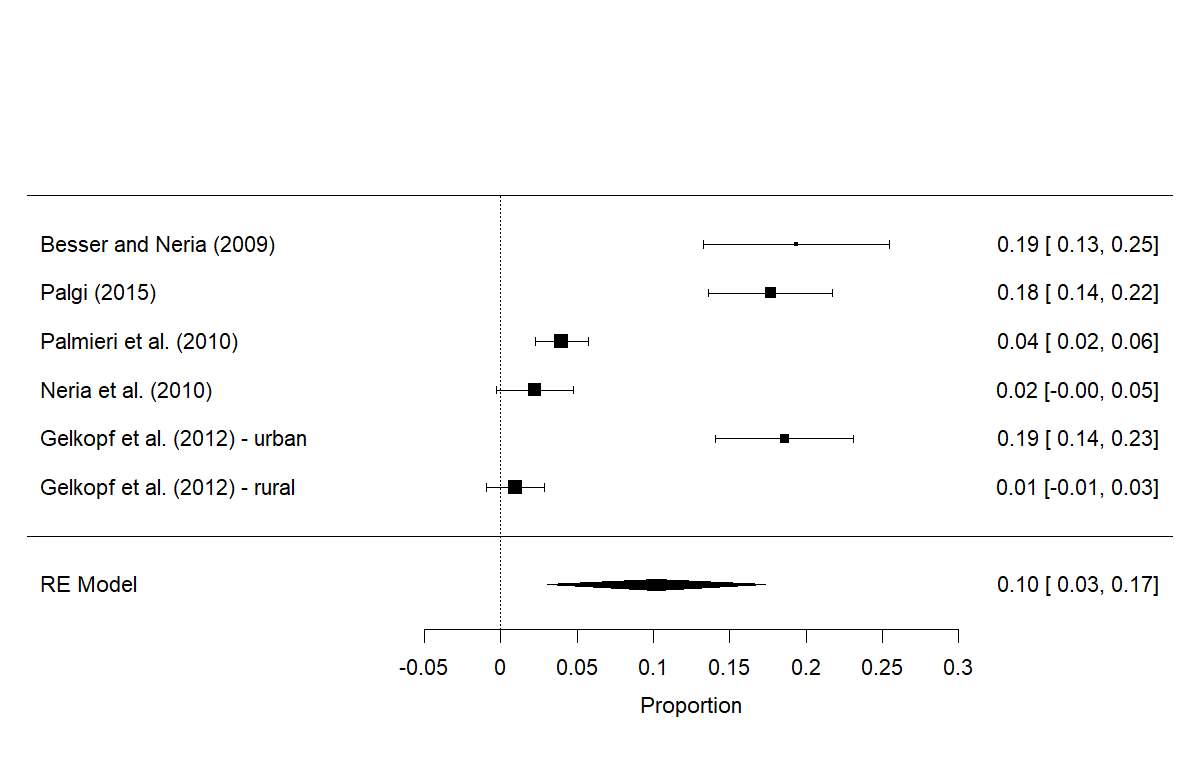


Indirectly affected community meta-analysis

**
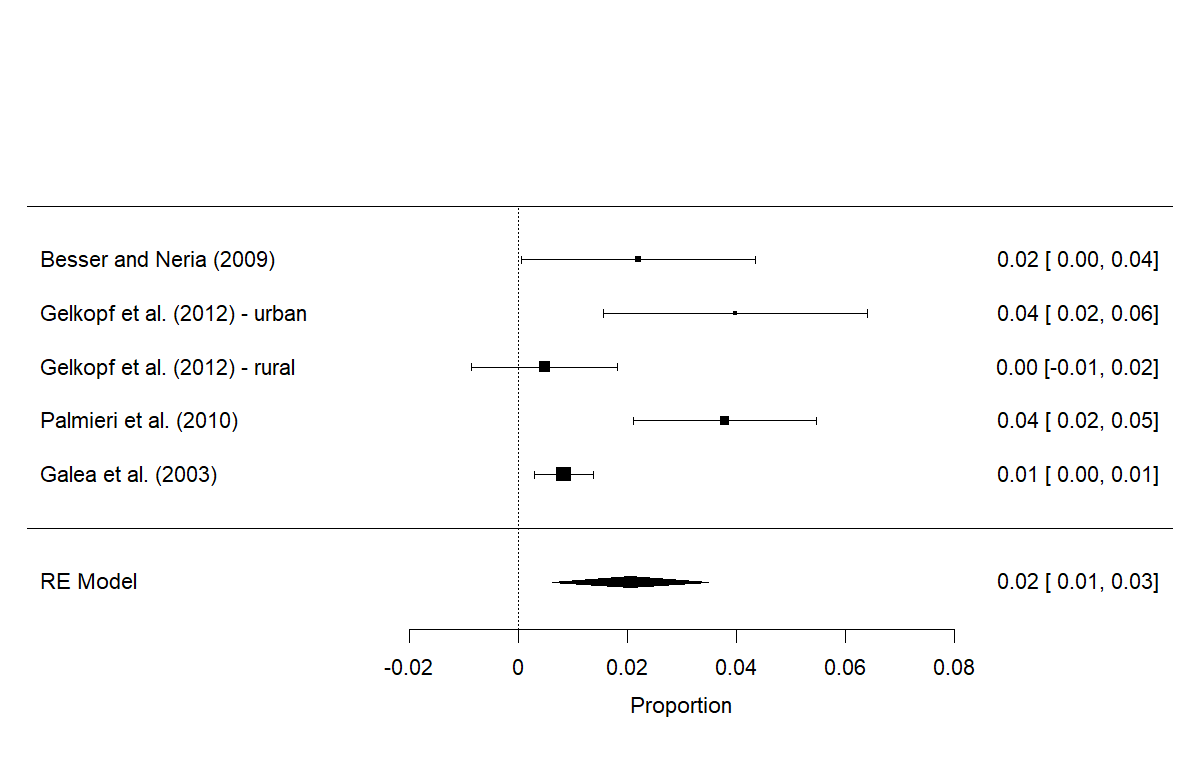
**

**Alternative Analysis: Ranges Based on Prediction Intervals**

As addressed in the methods section, there are alternative approaches to assessing the range of the expected prevalence of PTSD for the different groups. This alternative includes utilizing a prediction interval instead of a confidence interval (Spence & Stanley, 2016). This analysis was conducted using the function predict.rma (Viechtbauer, 2010). This function computes a prediction interval based on the results of the meta-analysis conducted for each group. Importantly, the estimates (which reflect the weighted mean effect sizes) remain the same, and the novelty of the function is in its prediction interval. As such, the expected numeric estimates for each group remain the same, and the difference is reflected in the ranges (i.e., prediction intervals). The prediction intervals reflect the lower and upper bound for prevalence estimates, and after multiplying by group size, a numeric range is determined. Results of the model with prediction intervals are presented in Table 1. Importantly, for most of the groups, the prediction lower bound of the prediction interval was lower than 0. In that case, the numeric estimate for the lower bound was set to 0. The results of the model indicate a range of 0.003%-14.8% of the population (in numerical estimates, 311-1,414,089 people) might develop PTSD in the aftermath of the war.

| 838,666 | 0 | 304,182 | 0.06 | -0.04-0.17 | 4,960,469 | **5) Civilians under moderate exposure to rocket attacks (living 40-80 km (25-50 miles) from the Gaza Strip)** |
| --- | --- | --- | --- | --- | --- | --- |
| 177,511 | 0 | 70,714 | 0.02 | -0.01-0.05 | 3,433,286 | **6) Indirectly affected communities (living more than 80 km (50 miles) from the Gaza Strip)** |
| 1,414,089 | 311 | 519,923 |  |  |  | **Total** |

Table 1: Results of the model using prediction intervals.

| **Upper bound N** | **Lower bound N** | **Expected N- PTSD** | **PTSD prevalence estimate** | **PTSD prevalence range** | **Group Size (after exclusions)** | **Exposure group** |
| --- | --- | --- | --- | --- | --- | --- |
| 24,816 | 311 | 12,564 | 0.32 | 0.01-0.63 | 39,664 | **1) Direct exposure to the October 7th terrorist attacks** |
| 34,676 | 0 | 12,354 | 0.10 | -0.08-0.29 | 121,061 | **2) Close proximity to the October 7th terrorist attacks** |
| 32,216 | 0 | 11,021 | 0.08 | -0.07-0.22 | 144,227 | **3) Soldiers in combat and support units involved in the war** |
| 306,204 | 0 | 109,088 | 0.10 | -0.08-0.29 | 1,069,011 | **4) Civilians under intense exposure to rocket attacks (living up to 40 kilometers (25 miles) from the Gaza Strip)** |

**REFERENCES**

Ankri, Y. L., Bachar, E., & Shalev, A. Y. (2010). Reactions to terror attacks in ultra-Orthodox Jews: The cost of maintaining strict identity. *Psychiatry: Interpersonal and Biological Processes*, 73(2), 190-197.

Barrett, D. H., Doebbeling, C. C., Schwartz, D. A., Voelker, M. D., Falter, K. H., Woolson, R. F., & Doebbeling, B. N. (2002). Posttraumatic stress disorder and self-reported physical health status among US Military personnel serving during the Gulf War period: a population-based study. *Psychosomatics*, 43(3), 195-205.

Barrett, D. H., Doebbeling, C. C., Schwartz, D. A., Voelker, M. D., Falter, K. H., Woolson, R. F., & Doebbeling, B. N. (2002). Posttraumatic stress disorder and self-reported physical health status among US Military personnel serving during the Gulf War period: a population-based study. *Psychosomatics*, 43(3), 195-205.‏

Besser, A., & Neria, Y. (2009). PTSD symptoms, satisfaction with life, and prejudicial attitudes toward the adversary among Israeli civilians exposed to ongoing missile attacks. *Journal of Traumatic Stress, 22*, 268 –275.

Besser, A., & Neria, Y. (2010). The effects of insecure attachment orientations and perceived social support on posttraumatic stress and depressive symptoms among civilians exposed to the 2009 Israel–Gaza war: A follow-up Cross-Lagged panel design study. *Journal of Research in Personality*, 44(3), 335-341.

Booth‐Kewley, S., Larson, G. E., Highfill‐McRoy, R. M., Garland, C. F., & Gaskin, T. A. (2010). Correlates of posttraumatic stress disorder symptoms in Marines back from war. Journal of Traumatic Stress: Official Publication of The International Society for Traumatic Stress Studies, 23(1), 69-77.‏

Boscarino, J. A., Adams, R. E., & Figley, C. R. (2004). Mental health service use 1-year after the world trade center disaster: Implications for mental health care*. General Hospital Psychiatry*, 26, 346–358

Browne, T., Hull, L., Horn, O., Jones, M., Murphy, D., Fear, N. T., ... & Hotopf, M. (2007). Explanations for the increase in mental health problems in UK reserve forces who have served in Iraq. *The British journal of psychiatry*, 190(6), 484-489.

Centers for Disease Control (1988). Vietnam Experience Study: health status of Vietnam veterans: psychosocial characteristics. *JAMA*, *259,* 2701–2707. [VES]Conejo-Galindo, J., Medina, O., Fraguas, D., Tera´n, S., Sainz-Corto´n, E., & Arango, C. (2008). Psychopathological sequelae of the 11 March terrorist attacks in Madrid: An epidemiological study of victims treated in a hospital. *European Archives of Psychiatry and Clinical Neuroscience, 258*, 28–34.

Dohrenwend, B. P., Turner, J. B., Turse, N. A., Adams, B. G., Koenen, K. C., & Marshall, R. (2006). The psychological risks of Vietnam for US veterans: a revisit with new data and methods. *Science*, 313(5789), 979-982.

Dohrenwend, B. P., Turner, J. B., Turse, N. A., Lewis‐Fernandez, R., & Yager, T. J. (2008). War‐related posttraumatic stress disorder in Black, Hispanic, and majority White Vietnam veterans: The roles of exposure and vulnerability. *Journal of Traumatic Stress*: Official Publication of The International Society for Traumatic Stress Studies, 21(2), 133-141.

Dolberg, O. T., Barkai, G., Leor, A., Rapoport, H., Bloch, M., & Schreiber, S. (2010). Injured civilian survivors of suicide bomb attacks: From partial PTSD to recovery or to traumatisation. Where is the turning point*? The World Journal of Biological Psychiatry, 11*, 344–351.

Du Preez, J., Sundin, J., Wessely, S., & Fear, N. T. (2012). Unit cohesion and mental health in the UK armed forces. *Occupational medicine*, 62(1), 47-53.

Du Preez, J., Sundin, J., Wessely, S., & Fear, N. T. (2012). Unit cohesion and mental health in the UK armed forces. *Occupational medicine*, 62(1), 47-53.‏

Eisen, S. A., Griffith, K. H., Xian, H., Scherrer, J. F., Fischer, I. D., Chantarujikapong, S., Hunter, J., True, W. R., Lyons, M. J. & Tsuang, M. T. (2004). Lifetime and 12-month prevalence of psychiatric disorders in 8,169 male Vietnam War era veterans. *Military medicine*, 169(11), 896-902.

Galea, S., Vlahov, D., Resnick, H., Ahern, J., Susser, E., Gold, J., ... & Kilpatrick, D. (2003). Trends of probable post-traumatic stress disorder in New York City after the September 11 terrorist attacks. *American journal of epidemiology*, *158*(6), 514-524.

Galea, S., Vlahov, D., Tracy, M., Hoover, D. R., Resnick, H., & Kilpatrick, D. (2004). Hispanic ethnicity and post-traumatic stress disorder after a disaster: Evidence from a general population survey after September 11, 2001. *Annals of Epidemiology*, 14, 520–531.

Gelkopf, M., Berger, R., Bleich, A., & Silver, R. C. (2012). Protective factors and predictors of vulnerability to chronic stress: A comparative study of 4 communities after 7 years of continuous rocket fire. *Social Science & Medicine, 74*, 757–766.

Gil, S., & Caspi, Y. (2006). Personality traits, coping style, and perceived threat as predictors of posttraumatic stress disorder after exposure to a terrorist attack: A prospective study. *Psychosomatic Medicine, 68*, 904–909.

Greene, T., Itzhaky, L., Bronstein, I., & Solomon, Z. (2018). Psychopathology, risk, and resilience under exposure to continuous traumatic stress: A systematic review of studies among adults living in southern Israel. *Traumatology*, *24*(2), 83.‏

Goldberg, J., Eisen, S. A., True, W. R., & Henderson, W. G. (1990). A twin study of the effects of the Vietnam conflict on alcohol drinking patterns. *American Journal of Public Health*, 80(5), 570-574.

Goldmann, E., Calabrese, J. R., Prescott, M. R., Tamburrino, M., Liberzon, I., Slembarski, R., ... & Galea, S. (2012). Potentially modifiable pre-, peri-, and postdeployment characteristics associated with deployment-related posttraumatic stress disorder among ohio army national guard soldiers. *Annals of epidemiology*, 22(2), 71-78.‏

Harbertson, J., Grillo, M., Zimulinda, E., Murego, C., Cronan, T., May, S., ... & Shaffer, R. (2013). Prevalence of PTSD and depression, and associated sexual risk factors, among male R wanda D efense F orces military personnel. *Tropical Medicine & International Health*, 18(8), 925-933.‏

Harbertson, J., Grillo, M., Zimulinda, E., Murego, C., Cronan, T., May, S., Brodine, S., Sebagabo, M., Araneta, M. R. G. & Shaffer, R. (2013). Prevalence of PTSD and depression, and associated sexual risk factors, among male Rwanda Defense Forces military personnel. *Tropical Medicine & International Health*, 18(8), 925-933.

Henriksen, C. A., Bolton, J. M., & Sareen, J. (2010). The psychological impact of terrorist attacks: Examining a dose-response relationship between exposure to 9/11 and axis I mental disorders. *Depression and Anxiety*, 27, 993–1000

Hobfoll, S. E., Canetti, D., Hall, B. J., Brom, D., Palmieri, P. A., Johnson, R. J., ... Galea, S. (2011). Are community studies of psychological trauma’s impact accurate? A study among Jews and Palestinians. *Psychological Assessment*, 23, 599–605.

Hoge, C. W., Auchterlonie, J. L., & Milliken, C. S. (2006). Mental health problems, use of mental health services, and attrition from military service after returning from deployment to Iraq or Afghanistan. *Jama*, 295(9), 1023-1032.

Hoge, C. W., Castro, C. A., Messer, S. C., McGurk, D., Cotting, D. I., & Koffman, R. L. (2004). Combat duty in Iraq and Afghanistan, mental health problems, and barriers to care. *New England journal of medicine*, 351(1), 13-22

Hotopf, M., Hull, L., Fear, N. T., Browne, T., Horn, O., Iversen, A., ... & Wessely, S. (2006). The health of UK military personnel who deployed to the 2003 Iraq war: a cohort study. *The lancet*, 367(9524), 1731-1741.

Ikin, J. F., Sim, M. R., Creamer, M. C., Forbes, A. B., McKenzie, D. P., Kelsall, H. L., Glass, D. C., McFarlane, A. C., Abramson, M. J., Ittak, P., Dwyer, T., Blizzard, L., Delaney, K. R., Horsley, K. W. A., Harrex, W. K.& Schwarz, H. (2004). War-related psychological stressors and risk of psychological disorders in Australian veterans of the 1991 Gulf War. *The British Journal of Psychiatry*, 185(2), 116-126.

Iversen, A. C., Fear, N. T., Ehlers, A., Hughes, J. H., Hull, L., Earnshaw, M., ... & Hotopf, M. (2008). Risk factors for post-traumatic stress disorder among UK Armed Forces personnel. *Psychological medicine*, 38(4), 511-522.‏

Iversen, A. C., Fear, N. T., Ehlers, A., Hughes, J. H., Hull, L., Earnshaw, M., Greenberg, N., Rona, R., Wessely, S. & Hotopf, M. (2008). Risk factors for post-traumatic stress disorder among UK Armed Forces personnel. *Psychological medicine*, 38(4), 511-522.

Iversen, A. C., van Staden, L., Hughes, J. H., Browne, T., Hull, L., Hall, J., ... & Fear, N. T. (2009). The prevalence of common mental disorders and PTSD in the UK military: using data from a clinical interview-based study. *BMC psychiatry*, 9(1), 1-12.‏

Jones, M., Rona, R. J., Hooper, R., & Wesseley, S. (2006). The burden of psychological symptoms in UK Armed Forces. *Occupational medicine*, 56(5), 322-328.

Jones, M., Rona, R. J., Hooper, R., & Wesseley, S. (2006). The burden of psychological symptoms in UK Armed Forces. *Occupational medicine*, 56(5), 322-328.‏

Jones, M., Sundin, J., Goodwin, L., Hull, L., Fear, N. T., Wessely, S., & Rona, R. J. (2013). What explains post-traumatic stress disorder (PTSD) in UK service personnel: deployment or something else?. *Psychological medicine*, 43(8), 1703-1712.‏

Kang, H. K., Natelson, B. H., Mahan, C. M., Lee, K. Y., & Murphy, F. M. (2003). Post-traumatic stress disorder and chronic fatigue syndrome-like illness among Gulf War veterans: a population-based survey of 30,000 veterans. *American journal of epidemiology*, 157(2), 141-148.

Kline, A., Ciccone, D. S., Weiner, M., Interian, A., St. Hill, L., Falca-Dodson, M., ... & Losonczy, M. (2013). Gender differences in the risk and protective factors associated with PTSD: a prospective study of National Guard troops deployed to Iraq. *Psychiatry: interpersonal & biological processes*, 76(3), 256-272.‏

Koenen, K. C., Harley, R., Lyons, M. J., Wolfe, J., Simpson, J. C., Goldberg, J., Eisen, S. A. & Tsuang, M. (2002). A twin registry study of familial and individual risk factors for trauma exposure and posttraumatic stress disorder. *The Journal of nervous and mental disease*, 190(4), 209-218.

Koenen, K. C., Stellman, J. M., Stellman, S. D., & Sommer Jr, J. F. (2003). Risk factors for course of posttraumatic stress disorder among Vietnam veterans: a 14-year follow-up of American Legionnaires*. Journal of consulting and clinical psychology*, 71(6), 980.‏

Kulka, R. A., Schlenger, W.E., Fairbank, J.A., et al (1990). Trauma and the Vietnam War generation: report of findings from the National Vietnam Veterans Readjustment Study. Brunner/ Mazel. [NVVRS]

Lawyer, S. R., Resnick, H. S., Galea, S., Ahern, J., Kilpatrick, D. G., & Vlahov, D. (2006). Predictors of peritraumatic reactions and PTSD following the September 11th terrorist attacks. *Psychiatry*, 69, 130–141

LeardMann, C. A., Smith, B., & Ryan, M. A. (2010). Do adverse childhood experiences increase the risk of postdeployment posttraumatic stress disorder in US Marines?. *BMC Public Health*, 10(1), 1-8.‏

Lee, H. A., Gabriel, R., Bolton, J. P. G., Bale, A. J., & Jackson, M. (2002). Health status and clinical diagnoses of 3000 UK Gulf War veterans. *Journal of the Royal Society of Medicine*, 95(10), 491-497.

Macera, C. A., Aralis, H. J., Highfill-McRoy, R., & Rauh, M. J. (2014). Posttraumatic stress disorder after combat zone deployment among Navy and Marine Corps men and women. *Journal of women's health*, 23(6), 499-505.

Macera, C. A., Aralis, H. J., Highfill-McRoy, R., & Rauh, M. J. (2014). Posttraumatic stress disorder after combat zone deployment among Navy and Marine Corps men and women. *Journal of women's health*, 23(6), 499-505.‏

MacGregor, A. J., Han, P. P., Dougherty, A. L., & Galarneau, M. R. (2012). Effect of dwell time on the mental health of US military personnel with multiple combat tours. *American journal of public health*, 102(S1), S55-S59.

MacGregor, A. J., Tang, J. J., Dougherty, A. L., & Galarneau, M. R. (2013). Deployment-related injury and posttraumatic stress disorder in US military personnel. *Injury*, 44(11), 1458-1464.‏

Maguen, S., Cohen, B., Cohen, G., Madden, E., Bertenthal, D., & Seal, K. (2012). Gender differences in health service utilization among Iraq and Afghanistan veterans with posttraumatic stress disorder. *Journal of Women's Health*, 21(6), 666-673.

Mayo, J. A., MacGregor, A. J., Dougherty, A. L., & Galarneau, M. R. (2013). Role of occupation on new-onset post-traumatic stress disorder and depression among deployed military personnel. *Military medicine*, 178(9), 945-950.

McCarren, M., Janes, G. R., Goldberg, J., Eisen, S. A., True, W. R., & Henderson, W. G. (1995). A twin study of the association of post-traumatic stress disorder and combat exposure with long-term socioeconomic status in Vietnam veterans. *Journal of traumatic stress*, 8, 111-124.‏

Miguel-Tobal, J. J., Cano-Vindel, A., Iruarrizaga, I., Gonza´lez-Ordi, H., & Galea, S. (2005a). Psychopathological repercussions of the March 11 terrorist attacks in Madrid. *Psychology in Spain*, 9, 75–80.

Nandi, A., Galea, S., Ahern, J., & Vlahov, D. (2005). Probable cigarette dependence, PTSD, and depression after an urban disaster: Results from a population survey of New York City residents 4 months after September 11, 2001. *Psychiatry*, 68, 299–310.

Neria, Y., Besser, A., Kiper, D., & Westphal, M. (2010). A longitudinal study of posttraumatic stress disorder, depression, and generalized anxiety disorder in Israeli civilians exposed to war trauma. *Journal of Traumatic Stress, 23*, 322–330.

North, C. S., Nixon, S. J., Shariat, S., Mallonee, S., McMillen, J. C., Spitznagel, E. L., ... Smith, E. M. (1999). Psychiatric disorders among survivors of the Oklahoma City bombing. *Journal of American Medical Association, 282*, 755–762.

North, C. S., Pfefferbaum, B., Kawasaki, A., Lee, S., & Spitznagel, E. L. (2011). Psychosocial adjustment of directly exposed survivors 7 years after the Oklahoma City bombing. *Comprehensive Psychiatry, 52*, 1–8.

North, C. S., Pfefferbaum, B., Narayanan, P., Thielman, S., McCoy, G., Dumont, C., ... Spitznagel, E. L. (2005). Comparison of post-disaster psychiatric disorders after terrorist bombings in Nairobi and Oklahoma City. *British Journal of Psychiatry, 186*, 487–493.

North, C. S., Pfefferbaum, B., Tivis, L., Kawasaki, A., Reddy, C., & Spitznagel, E. L. (2004). The course of posttraumatic stress disorder in a follow-up study of survivors of the Oklahoma City bombing. *Annals of Clinical Psychiatry, 16*, 209–215.

North, C. S., Pollio, D. E., Smith, R. P., King, R. V., Pandya, A., Surı´s, A. M., ... Pfefferbaum, B. (2011). Trauma exposure and posttraumatic stress disorder among employees of New York City companies affected by the September 11, 2001 attacks on the world trade center. *Disaster Medicine and Public Health Preparedness, 5*, 205–213.

Nuttman-Shwartz, O. (2014). Fear, functioning, and coping during exposure to a continuous security threat. Journal of Loss and Trauma, 19(3), 262-277.

Nuttman-Shwartz, O., Dekel, R., & Regev, I. (2015). Continuous exposure to life threats among different age groups in different types of communities. *Psychological Trauma: Theory, Research, Practice, and Policy*, 7(3), 269.

Ohtani, T., Twanami, A., Kasal, K., Yamasue, H., Kato, T., Sasaki, T., ... Kato, N. (2004). Post-traumatic stress disorder symptoms in victims of Tokyo subway attack: A 5-year follow up study. *Psychiatry and Clinical Neurosciences, 58*, 624–629.

O'toole, B. I., Marshall, R. P., Schureck, R. J., & Dobson, M. (1998). Posttraumatic stress disorder and comorbidity in Australian Vietnam veterans: risk factors, chronicity and combat. *Australian and New Zealand Journal of Psychiatry*, *32*(1), 32-42.Palgi, Y. (2015). Predictors of the new criteria for probable PTSD among older adults. *Psychiatry Research, 230*, 777–782.

Palmieri, P. A., Chipman, K. J., Canetti, D., Johnson, R. J., & Hobfoll, S. E. (2010). Prevalence and correlates of sleep problems in adult Israeli Jews exposed to actual or threatened terrorist or rocket attacks. *Journal of Clinical Sleep Medicine, 6*, 557–564.

Paz García-Vera, M., Sanz, J., & Gutierrez, S. (2016). A systematic review of the literature on posttraumatic stress disorder in victims of terrorist attacks. *Psychological reports*, *119*(1), 328-359.‏

Phillips, C. J., LeardMann, C. A., Gumbs, G. R., & Smith, B. (2010). Risk factors for posttraumatic stress disorder among deployed US male marines. *BMC psychiatry*, 10, 1-11.‏

Richardson, L. K., Frueh, B. C., & Acierno, R. (2010). Prevalence estimates of combat-related post-traumatic stress disorder: critical review. *Australian & New Zealand Journal of Psychiatry*, *44*(1), 4-19.‏

Riviere, L. A., Kendall-Robbins, A., McGurk, D., Castro, C. A., & Hoge, C. W. (2011). Coming home may hurt: risk factors for mental ill health in US reservists after deployment in Iraq. *The British Journal of Psychiatry*, 198(2), 136-142.‏

Rona, R. J., Jones, M., Sundin, J., Goodwin, L., Hull, L., Wessely, S., & Fear, N. T. (2012). Predicting persistent posttraumatic stress disorder (PTSD) in UK military personnel who served in Iraq: A longitudinal study. *Journal of psychiatric research*, 46(9), 1191-1198.

Rona, R. J., Jones, M., Sundin, J., Goodwin, L., Hull, L., Wessely, S., & Fear, N. T. (2012). Predicting persistent posttraumatic stress disorder (PTSD) in UK military personnel who served in Iraq: A longitudinal study. *Journal of psychiatric research*, 46(9), 1191-1198.‏

Sandweiss, D. A., Slymen, D. J., LeardMann, C. A., Smith, B., White, M. R., Boyko, E. J., ... & Millennium Cohort Study Team. (2011). Preinjury psychiatric status, injury severity, and postdeployment posttraumatic stress disorder. *Archives of general psychiatry*, 68(5), 496-504.‏

Shalev, A. Y., & Freedman, S. (2005). PTSD following terrorist attacks: A prospective evaluation. *American Journal of Psychiatry, 162*, 1188–1191.

Shear, K. M., Jackson, C. T., Essock, S. M., Donahue, S. A., & Felton, C. J. (2006). Screening for complicated grief among Project Liberty service recipients 18 months after September 11, 2001. *Psychiatric Services*, 57, 1291–1297

Smith, T. C., Ryan, M. A., Wingard, D. L., Slymen, D. J., Sallis, J. F., & Kritz-Silverstein, D. (2008). New onset and persistent symptoms of post-traumatic stress disorder self reported after deployment and combat exposures: prospective population based US military cohort study. *Bmj*, 336(7640), 366-371.

Stein, N. R., Schorr, Y., Krantz, L., Dickstein, B. D., Solomon, Z., Horesh, D., & Litz, B. T. (2013). The differential impact of terrorism on two Israeli communities. *American Journal of Orthopsychiatry*, 83(4), 528-535.

Stuber, J., Resnick, H., & Galea, S. (2006). Gender disparities in posttraumatic stress disorder after mass trauma. *Gender Medicine*, 3, 54–67.

Thompson, W. W., Gottesman, I. I., & Zalewski, C. (2006). Reconciling disparate prevalence rates of PTSD in large samples of US male Vietnam veterans and their controls. *BMC psychiatry*, 6, 1-10.

Toomey, R., Kang, H. K., Karlinsky, J., Baker, D. G., Vasterling, J. J., Alpern, R., ... & Eisen, S. A. (2007). Mental health of US Gulf War veterans 10 years after the war. *The British Journal of Psychiatry*, *190*(5), 385-393.Tracie Shea, M. T., Reddy, M. K., Tyrka, A. R., & Sevin, E. (2013). Risk factors for post-deployment posttraumatic stress disorder in national guard/reserve service members. *Psychiatry research*, 210(3), 1042-1048.

van Liempt, S., van Zuiden, M., Westenberg, H., Super, A., & Vermetten, E. (2013). Impact of impaired sleep on the development of PTSD symptoms in combat veterans: a prospective longitudinal cohort study. *Depression and anxiety*, 30(5), 469-474.‏

Wells, T. S., Ryan, M. A., Jones, K. A., Hooper, T. I., Boyko, E. J., Jacobson, I. G., ... & Gackstetter, G. D. (2012). A comparison of mental health outcomes in persons entering US military service before and after September 11, 2001. *Journal of traumatic stress*, 25(1), 17-24.‏

Xue, C., Ge, Y., Tang, B., Liu, Y., Kang, P., Wang, M., & Zhang, L. (2015). A meta-analysis of risk factors for combat-related PTSD among military personnel and veterans. *PloS one*, *10*(3), e0120270.‏

Zhang, G., North, C. S., Narayanan, P., Kim, Y., Thielman, S., & Pfefferbaum, B. (2013). The course of postdisaster psychiatric disorders in directly exposed civilians after the US Embassy bombing in Nairobi, Kenya: A follow-up study. *Social Psychiatry and Psychiatric Epidemiology, 48*, 195–203.
